# AI-driven neuroanalytic modeling for mental health: multichannel CNN-based autism spectrum disorder detection via facial pattern analysis

**DOI:** 10.3389/fncom.2026.1851416

**Published:** 2026-06-15

**Authors:** Narinder Kaur, Prabhdeep Singh, Kirandeep Singh, Jawad Khan, Dildar Hussain, Yeong Hyeon Gu, Reem Aljuaidi, Naif Waheb Rajkhan

**Affiliations:** 1 Department of Computer Science and Engineering, Chandigarh University, Mohali, India; 2 Department of Computer Science and Engineering, Graphic Era (Deemed to be University), Dehradun, India; 3 Department of Computer Science and Engineering, Chitkara University, Rajpura, India; 4 School of Computing, Gachon University, Seongnam, Republic of Korea; 5 Department of AI and Data Science, Sejong University, Seoul, Republic of Korea; 6 Department of Information System, College of Computer Engineering and Science, Prince Sattam Bin Abdalaziz University, Al-Kharj, Saudi Arabia; 7 Department of Computer Science, Faculty of Computing and Information Technology, King Abdulaziz University, Jeddah, Saudi Arabia

**Keywords:** autism spectrum disorder (ASD), brain disorder detection, computational neuroscience, facial pattern analysis, feature extraction, mental health diagnosis, neuroanalytic artificial intelligence

## Abstract

**Introduction:**

Autism Spectrum Disorder (ASD) is a neurodevelopmental condition characterized by abnormal brain connections, impaired cognitive functions, and dysfunctional behaviors, which, in mental health, is a major challenge to diagnose at an early age. Recent developments in Artificial Intelligence (AI) and computational neuroscience have made it possible to use neuroanalytic methods to identify subtle patterns related to brain disorders. Inspired by this, this study investigates facial pattern analysis as a non-invasive surrogate biomarker.

**Methods:**

A neuroanalytic deep-learning model is suggested on the basis of a Modified Histogram of Oriented Gradients-based Multichannel Convolutional Neural Network (MHMCNN). The technique comprises three steps, that is, (i) preprocessing and normalization of facial images, (ii) extraction of discriminative neuro-inspired features based on modified HOG descriptors, and (iii) multichannel CNN-based classification to discover complex structural and micro-pattern variations. The model is trained and tested on a publicly accessible facial autism dataset, and the performance of the model is tested using *k*-fold cross-validation.

**Results:**

The proposed MHMCNN framework achieved a validation accuracy of 98% and a test accuracy of 96.2%, demonstrating strong generalization capability for ASD facial image classification. The model attained a training accuracy of 99.8%, indicating effective feature learning during optimization. The combination of handcrafted feature descriptors and deep learning improves the feature representation and the strength of classification. Experimental findings support the enhanced generalization and stable recognition of ASD-related patterns.

**Discussion:**

The results emphasize the possible application of AI and computational neuroscience in neuroanalytic pattern detection in mental health diagnostics. The proposed solution offers a cost-effective and scalable solution to early screening of ASD by allowing observable facial characteristics to be related to underlying neurodevelopmental features. The work has helped in filling the gap between the phenotypic observations and the diagnosis of the disorder of the brain. Future studies will target the use of multimodal integration of neuroimaging and behavioral data to enhance understanding and clinical utility.

**Conclusion:**

This research introduces a new combination of AI and neuroanalytic principles to detect ASD that can further advance computational neuroscience-based mental health diagnostics. The suggested framework offers a scalable and affordable outcome of early screening and future expansion to multimodal frameworks of neuroimaging and behavioral data to increase clinical utility and interpretation.

## 1 Introduction

The World Health Organization estimates that ASD affects around one in every 100 children (Talantseva et al., 2023; Issac et al., 2025). Children affected with autism normally face symptoms of depression, anxiety, and ADHD. It is imperative that young children with this disorder undergo an autism evaluation as soon as possible in order to improve their social skills, communication difficulties, and lives. Researchers observed that early identification is critical for treatment and diagnosis of this illness (Thabtah, 2018). Building a model that is founded on the structural connections or exchanges between various brain regions is one of the most critical components of the search for neurological diseases such as autism, Alzheimer's disease, and epilepsy. In order to verify that an individual person has autism spectrum disorder (ASD), a comprehensive examination is required. This examination encompasses a comprehensive evaluation and a variety of assessments administered by licensed professionals, such as child psychologists. There are two prevalent methods for determining whether an individual has autism. The methods are named the Autism Diagnostic Interview Revised, commonly known as ADI-R, and the Autism Diagnostic Observation Schedule Revised, alias ADOS-R. However, despite their simplicity, they still necessitate a significant amount of time and effort (Arumugam et al., 2021; Bahathiq et al., 2022; Freeman et al., 1980). Individuals across all age groups may experience neurological and cognitive challenges due to genetic or environmental factors. It is feasible that the signals would exhibit substantial disparities in terms of their severity and their extent of dissemination. There are several distinctive indicators that can assist in determining whether an individual has this syndrome, including difficulty in communication, particularly in social settings; compulsions to repeat actions; persistent behavior, etc. These characteristics highlight the complexity of ASD and makes daily communication more difficult (Yahata et al., 2016; Wijesinghe et al., 2019; Plitt et al., 2015). The diagnosis of autism can be made based on a number of different symptoms. The vast majority of these symptoms are not particularly serious; nonetheless, a few of them might require the assistance of a specialist. There is a possibility that people who have an autism spectrum condition will have trouble communicating through their facial expressions, body language, vocalizations, etc. In addition, it is a condition that makes it difficult for a person to form relationships with other people with whom they interact. The growth of the brain is intimately linked to autism, which is a disorder that affects its development. In the context of mental health, the term “spectrum” refers to a collection of symptoms and behavioral patterns that display substantial variances from one another. This disorder can be identified in children as young as infants and as old as preschoolers (Hatim et al., 2025; Nogay and Adeli, 2024; Hasan et al., 2022). People who have ASD begin to display symptoms as early as childhood, and as a result, they struggle with social relationships, academic achievement, and employment opportunities (as mentioned earlier). The signs of autism often become apparent in youngsters during the first year of their lives. An unusual group of infants appears to develop normally during their first year; nevertheless, between the ages of 18 and 24 months, their behavior suffers a regression, and they begin to exhibit symptoms of autism (Abraham et al., 2017; Chen et al., 2011; Heinsfeld et al., 2018; Li et al., 2022). Studies abound demonstrating that children who receive medical attention before they age two typically have higher IQs than those who receive it following their 2-year anniversary. This is in contrast to children who do not begin receiving medical assistance until a much later point in their lives (Eslami et al., 2019; Thabtah and Peebles, 2019; Eslami et al., 2021). A child's ASD is discovered by means of several tests. These comprise the Screening Tool for Autism in Toddlers and Young Children (STAT), the Childhood Autism Rating Scale (CARS-2), and the Autism Spectrum Quotient (AQ) (Hossain et al., 2021). Early-stage diagnosis of this disease is extremely crucial since it has the potential to reduce or eliminate specific symptoms, thereby elevating quality standards of an individual. In spite of this, a considerable amount of time is lost as a consequence of the delay that occurs between the initial concern and the diagnosis. The application of machine learning techniques is essential for accelerating the entire process of diagnosis and making it easier for families to have access to intensive treatments in a timely manner. The speedy and reliable detection of individuals who may have autism spectrum disorder would not be the only benefit they would provide. The major concern of the proposed research is to design a model that uses machine learning to identify autism in young people. The aim of our study is to develop a machine learning model that can identify autistic children using behavioral observations, physical metrics, and language patterns. Herein, we automate and improve the testing model that can provide the best healthcare tool for the early detection of autism. This will improve ASD management and outcomes.

The main motivation behind autism detection is to identify autism spectrum disorder (ASD) at an early stage and enable individuals to get prompt intervention and finally enhance their quality of life. This initiative aims to enhance autism awareness and improve the quality of life for autistic individuals and their families by reducing the identification and service inequities. Our key contributions are given as below:

- In this study, a machine learning-based autism spectrum disorder (ASD) model for early disease detection is proposed to predict or classify autism disorder in children as either autistic or non-autistic.
- It is a three-step model in which the data supplied at first, data preprocessing is performed, and proposed HOG descriptors were added. In the third step, MHMCNN was applied.
- The proposed model empowers the families and medical professionals by addressing autism-related public health challenges and improving autistic youths' quality of life.
- The experimental results show that the proposed model, which is mainly based on multichannel CNN and HOG methodology, is efficient as compared to the state-of-the-art models.

This paper is divided into several sections. Section 2, which follows the introduction, will discuss related works. Section 3 of this document will firstly discuss the dataset, followed by detailed methodology that is used in this study. Section 4 of this document also describes the outcomes of the experiments. Section 5 with a discussion of future possibilities.

## 2 Literature review

In Niu et al. (2020), an 809-subject multichannel DANN model on the ABIDE repository achieved 73.2% accuracy in the classification of autism by incorporating three brain MRIs and personal characteristics. Munandar et al. (2018) predicts the sentiments about the topics collected from the number of tweets by applying multichannel CNN and achieves a low loss error and high accuracy of 99.17%. Li et al. (2022) proposes an automated method for the autism classification using MRI images by applying multiple transformed data, yielding high accuracy results. Zhang et al. (2022) proposes a multichannel network with dense attention to extracting features from images with low resolution. Shi et al. (2022) was suggesting a multichannel CNN and data enhancement for recognizing human behavior in wearable tech, which leads to a better detection effect. Ma et al. (2023) suggests the use of DC-CNN, which is a CNN that has two channels and a pool of attention, in order to identify fake news. Similarly, Samadi and Momtazi (2022) introduced a multichannel CNN model using three unique embedding channels, which helps in detecting the fake news applied to the fake news dataset on COVID-19. Trivedi et al. (2019) proposed a multichannel CNN architecture for the recognition of facial expressions using different image filtering and different edge detection techniques. CNN classifier was applied on different images to classify mixed gas (Oh et al., 2024). Deep learning model was proposed that is capable of locating seizures in multichannel EEG data that are brought on by epilepsy rather readily (Craley et al., 2021). Employing a consolidated deep learning model, the author suggests an automated way for detecting hostile behavior in the form of cyberbullying (Alotaibi et al., 2021). This method classifies comments taken from Twitter into aggressive and non-aggressive. Deploying multichannel deep learning using BiGRU, the transformer block, and the CNN, the performance of the proposed technique was evaluated to have an accuracy of 88%. Recent research has shown growing interest in applying machine learning and deep learning techniques for early detection of Autism Spectrum Disorder (ASD). For instance, Hatim et al. provided a comprehensive overview of existing approaches, highlighting that deep learning models, particularly CNN-based methods, tend to outperform traditional techniques (Hatim et al., 2025). However, they also pointed out ongoing challenges such as limited dataset diversity and lack of model interpretability. Similarly, Rasul et al. (2024) conducted a comparative evaluation of several machine learning models using healthcare datasets. Their results showed that ensemble and neural network-based methods consistently achieve better accuracy than conventional classifiers, especially when trained on well-balanced data. In another study, Farooq et al. (2023) explored ASD detection across both children and adults using machine learning models. Their findings emphasized the importance of effective preprocessing and feature selection in achieving high classification performance. Earlier work by Liao et al. (2022) demonstrated that machine learning techniques such as decision trees and SVM can support early ASD diagnosis using clinical and behavioral data. However, their study also highlighted that model performance is highly dependent on the quality and representativeness of the dataset. Some of the existing ASD detection methods are discussed below:

**Parental interviews:** since parents are typically the first to notice a change in their child's behavior, these interviews are crucial (Bosl et al., 2018). Standardized questionnaires used for screening like the ASQ-3, the Modified Checklist for Autism in Toddlers, M-CHAT, and Social Communication questionnaires (SCQ).

**Observations made on behavior:** observations made on behavior is another form of evaluation that psychiatrists employ to evaluate children suspected of having autism spectrum disorder. The Behavior Observation Scale (BOS) is a tool that helps evaluate children who have ASD (Chen et al., 2011). It has 67 objectively described behaviors. Various varieties of autism and different ages each have their own unique sets of objective behaviors that are measured by the BOS.

**Analysis of the electroencephalogram (EEG):** EEG analysis is a clinical procedure that is utilized for the early detection of ASD at the young age of 24 months (Bone et al., 2016). The anatomy of the brain, as well as the connectivity of its cerebral pathways, may be analyzed using EEG. Within the first year of life, a child's brain has reached its maximum potential.

**Magnetic resonance imaging (MRI) processing:** an MRI evaluation might be utilized to locate issues that have been brought to light in the brain. The use of structural MRI in the diagnosis of structural brain abnormalities is helpful (Geetha Ramani and Sivaselvi, 2017).

## 3 Materials and proposed methodology

### 3.1 Materials

#### 3.1.1 Data set

The dataset used in this study is the Autistic Children Facial Dataset, which is publicly available on Kaggle (2020). This dataset is widely used for automated Autism Spectrum Disorder (ASD) detection based on facial image analysis. It contains 2,936 images of the features of children with and without autism as described in Table 1. The dataset is organized into class-wise folders, where each class contains corresponding facial images. However, it is important to note that the dataset does not provide detailed demographic metadata such as age distribution, gender, ethnicity, or imaging acquisition conditions (e.g., camera type, lighting variations, or clinical settings). This lack of demographic and acquisition information may introduce potential bias and limit the generalizability of the proposed model across diverse populations. Future work will focus on validating the model using multi-source datasets with richer metadata. In many implementations, the dataset is further divided into training, validation, and testing subsets, maintaining class balance. Before training, all images are typically resized to a fixed dimension (e.g., 224 × 224 pixels), Normalized for pixel intensity, and optionally augmented (rotation, flipping, and brightness adjustment) to improve generalization. These preprocessing steps ensure compatibility with deep learning models such as CNNs and transfer learning architectures.

**Table 1:** Dataset split for training, validation, and testing.

Dataset split	Autistic	Non-autistic	Total
Training	1,268	1,268	2,536
Validation	100	100	200
Testing	100	100	200
Total	1,468	1,468	2,936

**Figure 1:**
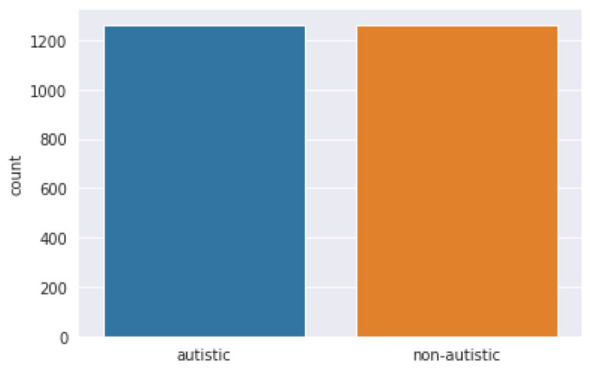
Class distribution of the training dataset (2,536 images), showing balanced samples of autistic (1,268) and non-autistic (1,268) classes (Kaggle, 2020).

**Figure 2:**
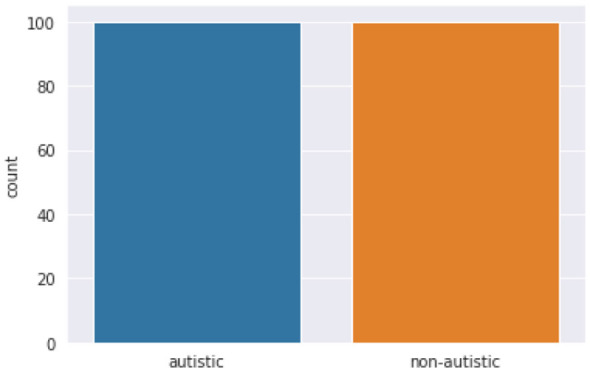
Class distribution of the validation dataset (200 images), consisting of 100 autistic and 100 non-autistic samples.

**Figure 3:**
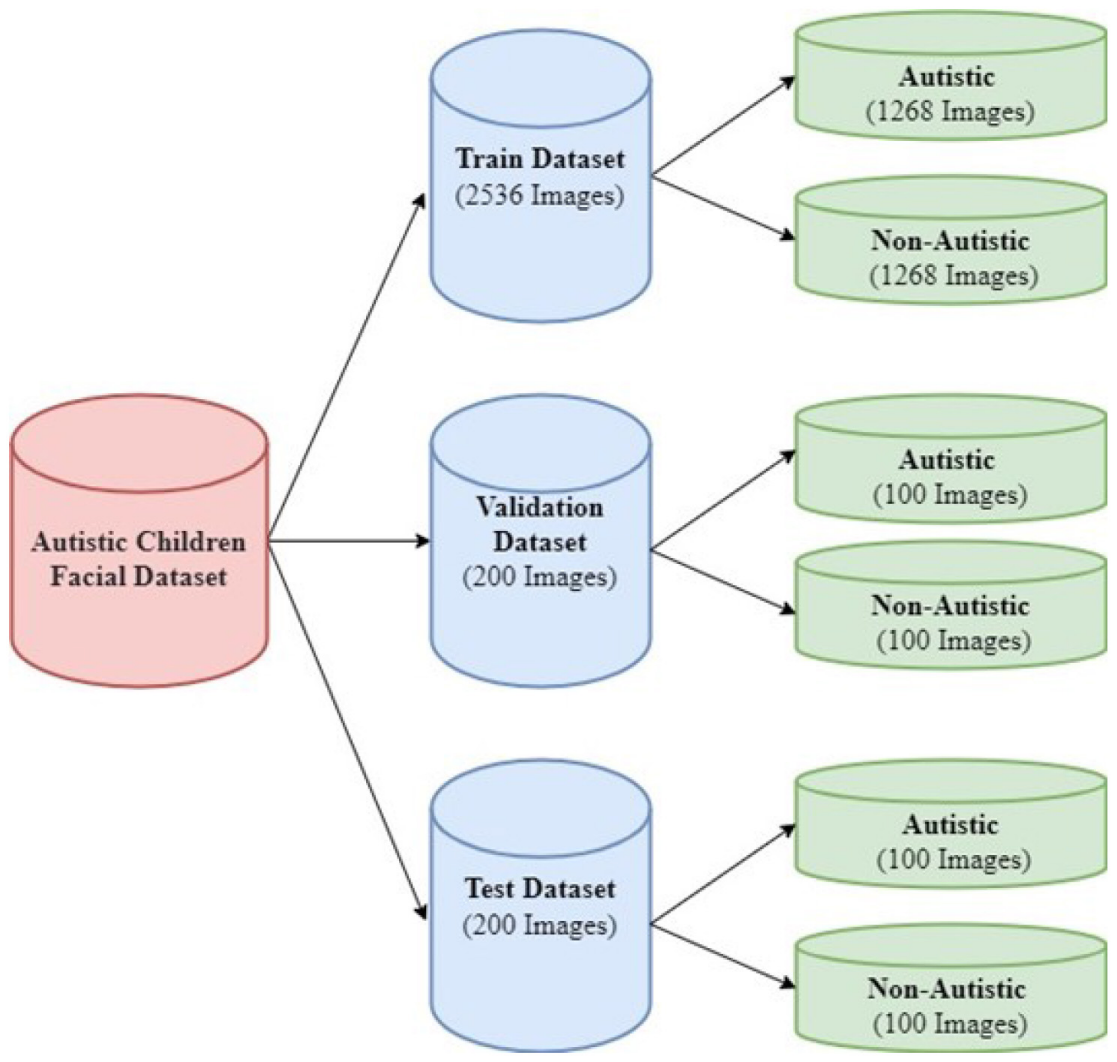
Description of facial data-set of autism.

In this dataset, Train, Valid, and Test are the three categories presented in which the entire dataset is divided. The images of the faces of autistic and non-autistic infants are contained in two subfolders within each folder. The first training data set comprises 1,200 images of children with autism as shown in Figure 1 and without autism as shown in Figure 2. Secondly, the test data set has 100 facial images of both types in different folders. Finally, the third valid data set comprises 50 autistic images and 50 non-autistic children's images. The description of the Facial set of Autism available on Kaggle is as per Figure 3. The test data set has 100 facial images of both types in different folders. The valid data set comprises facial images of 50 autistic and 50 non-autistic children's images. The description of the facial dataset of autism available on Kaggle is as per Figure 3.

#### 3.1.2 Data preprocessing

In this phase, the data preprocessing of images is performed using deep learning and improves the performance of a model by making the data more suitable for training. As the images in a dataset may have different sizes, this can cause problems during training. Therefore, resizing all the images to the same size can be helpful. The most common sizes for images are 224 × 224, 256 × 256, or 299 × 299 pixels. Initially, all input images are resized to 128 × 128 and converted to grayscale for efficient HOG-based feature extraction. The extracted HOG representations are then transformed into a suitable format and resized to 224 × 224 to serve as input to the multichannel CNN model. The visualization of the HOG descriptor actually visualizes the gradients of the image. Later, a color channel has been added to every image of the data set as it got removed while converting facial images into HOG images. Thus, the preprocessing pipeline follows a sequential flow:

Raw Image → *Resize* (128 × 128) → *HOG Feature Extraction*→*Resize* (224 × 224) → *Data Augmentation*→*CNN Input*

#### 3.1.3 Data augmentation

After the data is collected or gathered, there is a requirement of image augmentation because the data set comprises images with different shapes and sizes. Data augmentation of an image is a method for intentionally raising the size of a dataset used for training. Artificial training pictures are generated via image augmentation using diverse techniques, such as random rotation, shifts, shears, flips, etc. The normalization and resizing are applied to all the images. In this phase of the framework, every image of the dataset is resized into (224 × 224) pixels. This resizing is done as it helps to reduce the computational cost and processing time. All images are then transformed from RGB images to grayscale images using OpenCV, and using Keras, the images are converted into NumPy arrays. Once done with conversion, the proposed framework for the classification of autism is applied to the dataset, which will be discussed in next section.

#### 3.1.4 HOG-feature descriptor and extraction

An image has a lot of information incorporated with it, like color, shapes, background, intensity, edges, and many more. Histogram of Oriented Gradients, or HOG for short, is a type of feature descriptor that may be applied to input picture in order to extract desired features from that picture. One of the algorithms that takes an image as its input and produces either a feature array or a feature vector as its output is called a feature descriptor. The main goal of HOG is not only to work on edges of the picture but also uses direction, that is, Gradient (magnitude) and orientation (direction) of the edges. The HOG algorithm first calculates Gradient (magnitude) and orientation (direction) of the edges and creates the histogram on the basis of these gradients and orientations. Then, Histogram is normalized to generate the HOG features. The input image is then broken into smaller portions which are known as “localized portions.” For each portion Gradient and orientation is calculated and Histogram of each region is generated. The key parameters of the HOG descriptor are orientations, pixels per cell, and cells per block, which helps in controlling the dimension of the feature vector (output). For Every image, Small patches are taken as shown in Figure 2. There is a specified pixel matrix for each patch. New gradient for both the *X* and *Y* directions is computed for every pixel. As a result, Two new metrics will be generated to store gradients in the *X* and *Y* directions, respectively. Following is the operation used to obtain the gradient of image (*I*). *r* and *c* are the row and column of every pixel of an image as described in Equations 1–5.


$$\begin{array}{l} f_{X}=I\left(r,c+1\right)-I\left(r,c-1\right) \end{array}$$ (1)

$$\begin{array}{l} f_{Y}=I\left(r-1,c\right)-I\left(r+1,c\right) \end{array}$$ (2)

This process will be repeated for all the pixels of the image. After calculating gradients, magnitude and direction for each pixel will be determined using Pythagoras theorem. For an image *I*(*X, Y*), the following is the formula used to calculate gradients. After calculating gradients, magnitude and direction for each pixel will be determined using the Pythagorean theorem.


$$\begin{array}{l} Magnitude=\sqrt{{f_{X}}^{2}+{f_{Y}}^{2}} \end{array}$$ (3)

$$\begin{array}{l} Orientation=tan\left(\emptyset \right)=\frac{f_{y}}{f_{x}} \end{array}$$ (4)

$$\begin{array}{l} \emptyset =atan\frac{f_{y}}{f_{x}} \end{array}$$ (5)

Using these gradients and magnitude, Histogram will be generated. Histogram represents the frequency of these orientations, which is calculated for nine angles (0°, 20°, 40°, 60°, 80°, 100°, 120°, 140°, and 160°). For every pixel, orientation is calculated and frequency of orientation is stored in the form of (9 × 1) matrix.

## 4 Proposed model

### 4.1 System model

**Figure 4:**
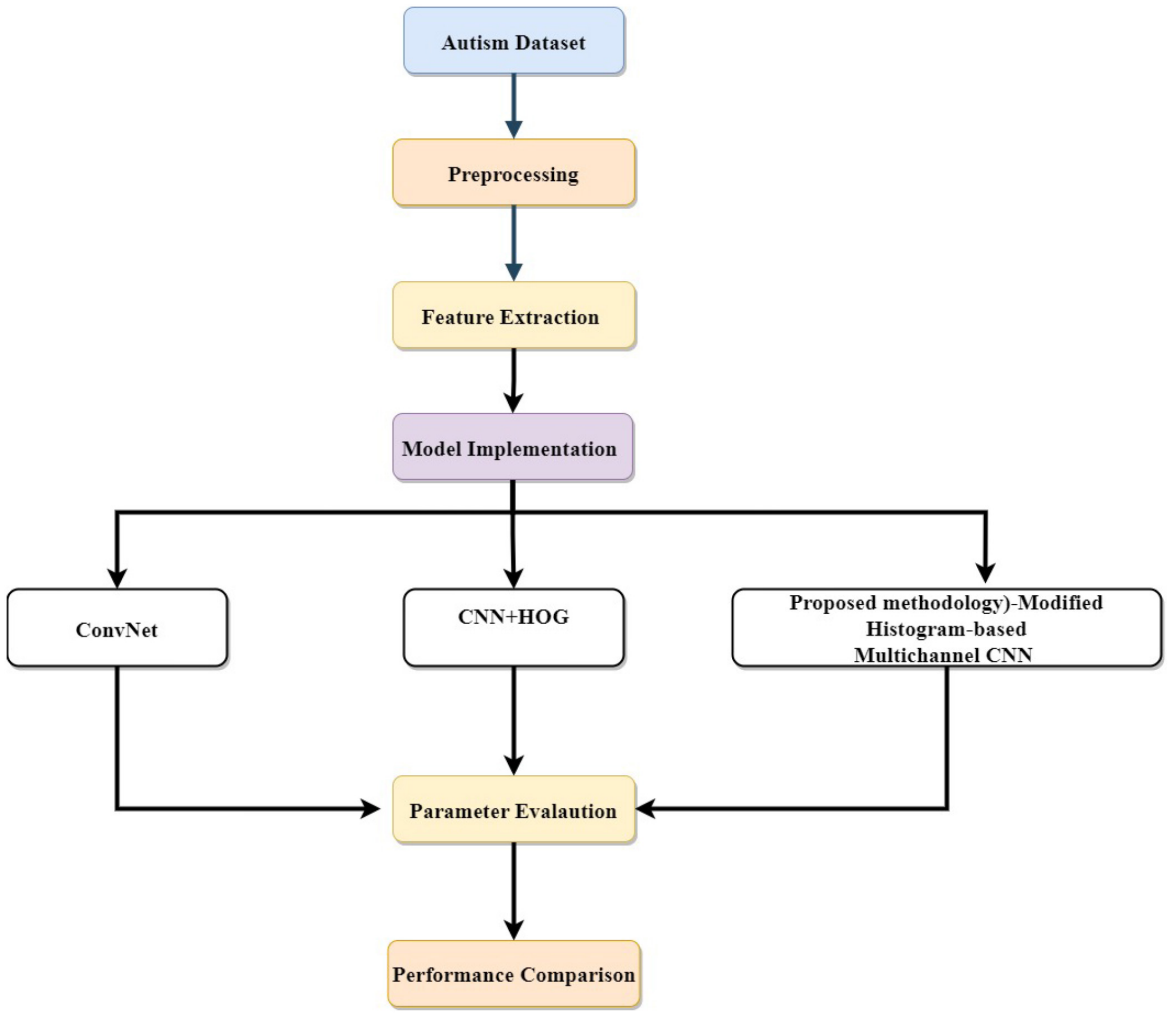
Proposed system architecture.

This section describes our proposed model or system architecture. At first, the autism dataset is applied. The preprocessing was performed, and the key features were extracted. In the third phase named model implementation was performed. In this phase, we applied our proposed modified histogram of gradient. The output features are then trained to classify the detection of autism. The process of applying the HOG descriptor is shown in Figure 4. This includes data collection, feature extraction, sampling, model creation, and ASD classification.

### 4.2 Architecture and working mechanism

**Figure 5:**
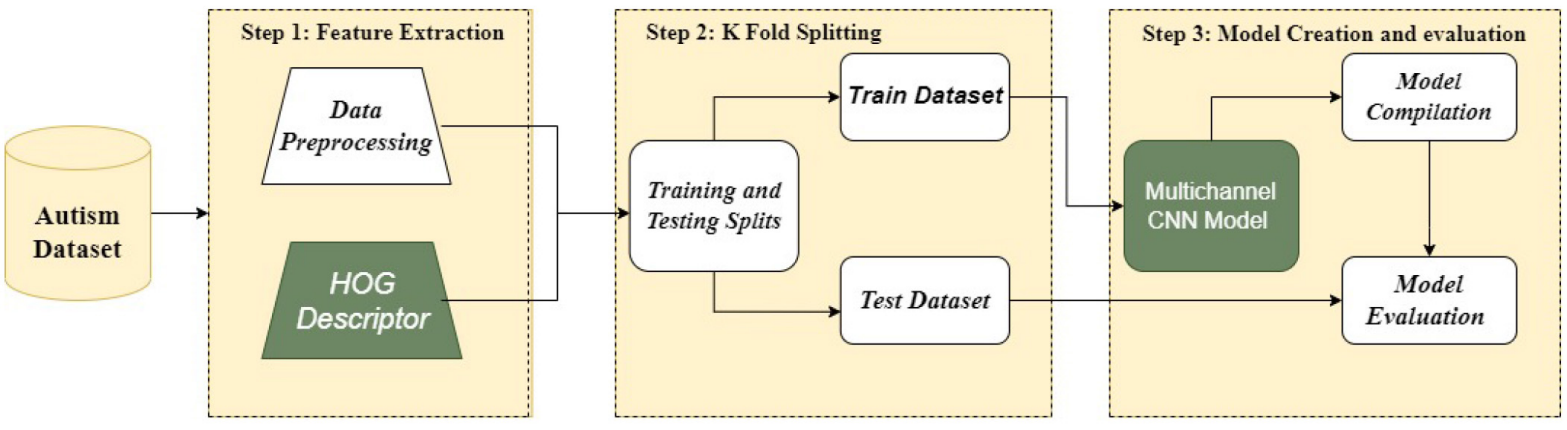
Flow Diagram of model.

In this section, we present the working mechanism of our proposed model. Figure 5 shows the flow diagram of our proposed model. In this figure, at first the input is applied using the AUTSIM dataset. In step 1, the feature extraction is performed. In this step, the data preprocessing is performed, and the HOG descriptor is applied using Algorithm 1. The massive dataset is acquired to train, test, and validate. Since the images are colored facial images, these are converted into grayscale. Resizing of images into (128 × 128) is done for better feature extraction. Resizing of images into (128 × 128) is done for better feature extraction. For every image, the gradient for both the *X* and *Y* directions is computed for every pixel. Following is the operation used to obtain the gradient of the image (*I*). *r* and *c* are rows and columns of every pixel of an image as described in Equations 6–10.


$$\begin{array}{l} f_{X}=I\left(r,c+1\right)-I\left(r,c-1\right) \end{array}$$ (6)

$$\begin{array}{l} f_{Y}=I\left(r-1,c\right)-I\left(r+1,c\right) \end{array}$$ (7)

Algorithm 1 *ModifiedHOG*(*get*−*data*). 
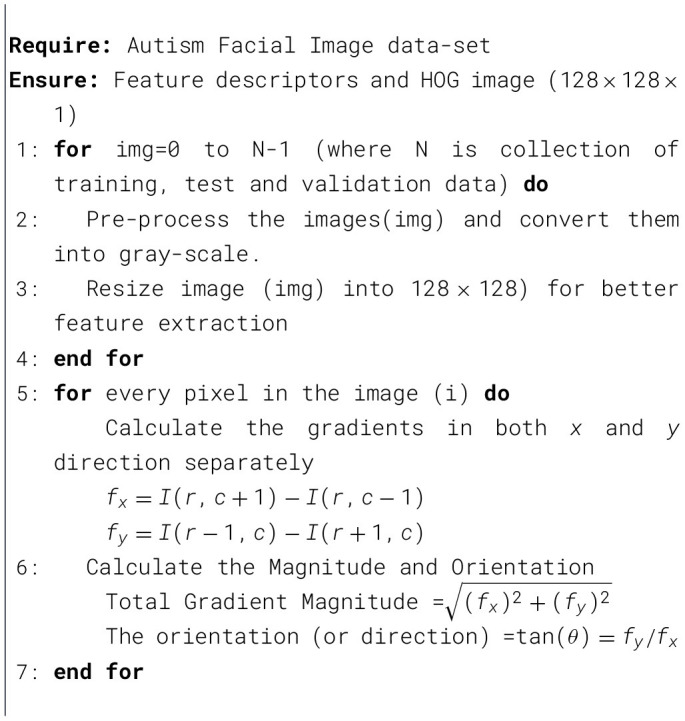

After calculating gradients, magnitude, and direction, for each pixel, it will be determined using the Pythagorean theorem.


$$\begin{array}{l} Magnitude=\sqrt{{f_{X}}^{2}+{f_{Y}}^{2}} \end{array}$$ (8)

$$\begin{array}{l} Orientation=tan\left(\emptyset \right)=\frac{f_{y}}{f_{x}} \end{array}$$ (9)

where


$$\begin{array}{l} \emptyset =atan\frac{f_{y}}{f_{x}} \end{array}$$ (10)

Using these gradients and magnitudes, a histogram will be generated. The histogram represents the frequency of these orientations, which is calculated for nine angles (0°, 20°, 40°, 60°, 80°, 100°, 120°, 140°, and 160°). For every pixel, orientation is calculated, and the frequency of orientation is stored in the form of a (9 × 1) matrix. In order to get a modified version of HOG, some of the HOG descriptors have been changed. It will get deeper into the images for better extraction of the features. The author has made direct use of the hog function that may be found in the “skimage.features” file. Therefore, gradients, magnitude (total gradient), and direction need not be calculated separately. The function would calculate it internally and return the feature matrix. In addition, if the option visualize = True is supplied, a picture of the HOG is returned.

In the second step, Algorithm 2 is applied. In Algorithm 2, at first the image read action is performed, and a batch of 64 with dimensions of 224 by 224 by 3, which measures width, height, and channel. A call to the modified HOG function is executed for each image. It is time to determine the filter and kernel dimensions. In the last stage, CNN layers are included and then amalgamated. Add 0.5 to the dropout rate. Employ the activation function ReLU. The proposed model has been refined with the Adam optimizer. The loss on the prediction may be computed. Feature arrays from images are extracted using CNN and HOG techniques. The main reason for combining HOG with CNN is the powerful combination in terms of computational cost and training time. It overcomes the disadvantage of CNN, which is to take ample time in training the model, where the combination of both takes a few minutes rather than a few hours. The major advantage of HOG is a balanced trade-off between complexity and accuracy. Combined features retrieved from HOG and CNN are then fed into the model to train the classification model. This will lead to the generation of large numbers of features, which will help to diagnose the autism. The model is then validated using the sigmoid function learning technique. The performance of the trained model is determined using the number of parameters like accuracy, loss, confusion matrix, etc. The test images are tested on this model to classify them into autistic and non-autistic images.

Algorithm 2 MHMCCNET.

**Require:**  Image with HOG feature
**Ensure:**  
  1:  Read the image in a batch of 64 of size (224 ×
    224 × 3), i.e., (Width, Height, and Channel).
  2:  **for** each image i in (*x*_*i*_, *y*_*i*_) **do**
  3:   CALL Modified HOG (*x*_*i*_, *y*_*i*_)
  4:  end **for**
  5:  Initialize filter (f) and Set kernel size (k) = 3
  6:  **while** *f* ≤ 128 **do**
  7:   Add CNN layers (k,f)
  8:   Set *f* = *f* × 2
  9:  **end while**
10:  Merge All CNN layers
11:  Add dropout rate=0.5
12:  Apply Relu activation *R*(*a*_*i*_) = *max*(0, *a*_*i*_)
        where *a*_*i*_ is the input vector
13:  Compile Model with Optimizer=adam
14:  Find the loss on the prediction as per binary
     cross entropy
          Loss=-(y log(p) + (1-y) log(1-p)
          where y is the binary indicator (0 for
     autism, 1 for non-autistic) and p is the predicted
     probability for class
15:  Evaluate model
16:  Output: [P(autism), P(non-autistic)] where P is
     the probability of the class.

#### 4.2.1 Comparison between standard HOG and modified HOG

The Histogram of Oriented Gradients (HOG) is a widely used feature descriptor that captures local shape information by computing gradient orientations and their distributions. While the standard HOG formulation focuses on fixed gradient computation and histogram binning, the proposed Modified HOG (MHOG) introduces several enhancements to improve feature representation for ASD facial pattern analysis. In standard HOG, gradients are computed using fixed operators, followed by orientation binning (typically nine bins) and block normalization. The extracted features are then directly used for classification. In contrast as described in Table 2, the proposed MHOG modifies the conventional pipeline in the following ways:

1. **Preprocessing enhancement:** all facial images are resized to a uniform dimension (128 × 128) and converted to grayscale to ensure consistency and reduce computational complexity.
2. **Feature representation:** instead of using HOG features as standalone descriptors, the MHOG output is transformed into structured feature maps that retain spatial information, making them compatible with CNN input.
3. **Integration with deep learning:** the extracted HOG features are not directly classified; instead, they are fed into a multichannel CNN architecture, enabling hierarchical feature learning and improved pattern recognition.
4. **Computational optimization:** the use of built-in optimized HOG functions (e.g., skimage.feature.hog) allows efficient gradient and orientation computation without manual implementation, reducing computational overhead.
5. **Multi-channel feature fusion:** the MHOG features are processed across multiple CNN channels, allowing the model to learn diverse representations from the same input.

**Table 2:** Comparison between standard HOG and proposed modified HOG (MHOG).

Aspect	Standard HOG	Modified HOG (proposed)
Gradient computation	Fixed (manual implementation)	Optimized (library-based)
Image preprocessing	Optional	Standardized resizing + grayscale
Orientation bins	Fixed (e.g., nine bins)	Same (retained)
Feature output	1D feature vector	Structured feature map
Spatial information	Partially preserved	Better preserved for CNN input
Normalization	block normalization	Implicit via CNN layers
Classification	Traditional ML models	Multichannel CNN
Feature learning	Handcrafted only	Hybrid (HOG + deep learning)
Computational efficiency	Moderate	Improved via optimized functions

These modifications enhance the discriminative capability of HOG by combining handcrafted feature extraction with deep feature learning. Hyper-parameter values for predefined and proposed models. are described in Table 3.

**Table 3:** Hyper-parameter values for predefined and proposed models.

Hyper-parameters	CNN	HOG + CNN	Proposed
Input shape	(150, 150, 1)	(48, 48, 1)	(48, 48, 1)
Feature extraction	–	HOG	HOG
Epochs	50	50	50
Hidden layers	1	1	Channel 1–1 Channel 2–1 Channel 3–1
Activation function	Output layer: Sigmoid; Hidden layer: ReLU		
Optimizer	Adam	Adam	Adam
Loss function	Binary cross entropy	Binary cross entropy	Binary cross entropy
Metrics	Accuracy, validation accuracy, loss, validation loss	Accuracy, validation accuracy, loss, validation loss	Accuracy, validation accuracy, loss, validation Loss
Learning rate	–	–	0.001
Batch size	32	32	64

The novelty of the proposed MHOG lies not in altering the fundamental gradient formulation of HOG, but in restructuring its output and integrating it with a multichannel CNN framework to enable enhanced feature learning and improved classification performance.

## 5 Experimentation, results, and detailed analysis

### 5.1 Experimental setup

The experiment was done on a variety of hardware devices and Python (Python Software Foundation, Wilmington, Delaware, USA) library settings so that an autism detection system (ADS) could be made. All experiments are conducted using Python using Google Colaboratory on a PC, which is 11th Gen Intel^(*R*)^ Core^*TM*^ i5-1155G7 with a CPU of 2.50 GHz and 8 GB RAM. Python libraries used for the conduct of this work are the TensorFlow library, Keras library, Panda Seaborn, Matplotlib, and Numpy. Training time for the proposed method was actually 1 h, including feature extraction, and testing time was approximately 7–8 min. The detailed result is discussed in the next section.

### 5.2 Performance metrics

In order to assess the performance of any prediction model on test data, we have implemented a variety of performance metrics, such as Accuracy, Precision, Recall, and *F*1-score as described in Equations 11–15 respectively (Nielsen et al., 2013; Akter et al., 2019; Chaddad et al., 2017). In this context, the execution time serves as a representation of the computation time necessary for algorithm training. Other performance metrics are classified into four categories: true positive, true negative, false positive, and false negative. These categories are primarily determined by the comparison of predicted and actual values from the training set. A true positive is a situation in which the true value and the predicted value are both positive. A true negative is a situation in which the true value and the predicted value are both negative. A false positive is a situation in which the observation is negative and the predicted value from training is positive. A false negative is a situation in which the true value is positive and the predicted value is negative (Heinsfeld et al., 2018; Kazeminejad and Sotero, 2019). Based on the above evaluations, the performance measures are as follows:


$$\begin{array}{c} Accuracy=\frac{TruePositive+TrueNegative}{TruePositive+TrueNegative+FalsePositive+FalseNegative} \end{array}$$ (11)

$$\begin{array}{c} Precision=\frac{TruePositive}{TruePositive+FalsePositive} \end{array}$$ (12)

$$\begin{array}{c} Recall=\frac{TruePositive}{TruePositive+FalseNegative} \end{array}$$ (13)

$$\begin{array}{c} Specificity=\frac{TrueNegative}{TrueNegative+FalsePositive} \end{array}$$ (14)

$$\begin{array}{c} F1Score=2*\frac{Precision\times Recall}{Precision+Recall} \end{array}$$ (15)

### 5.3 Results

In this section, we compared our proposed model along with the CNN and CNN + HOG models. The CNN and CNN + HOG are baseline methods. we have applied the same facial dataset on these and our proposed model using special feature extraction. The proposed model gives better accuracy than baseline methods.

#### 5.3.1 ConvNet

**Figure 6:**
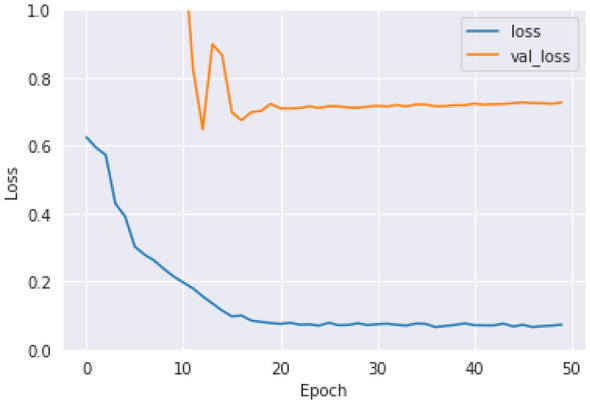
Loss of CNN.

**Figure 7:**
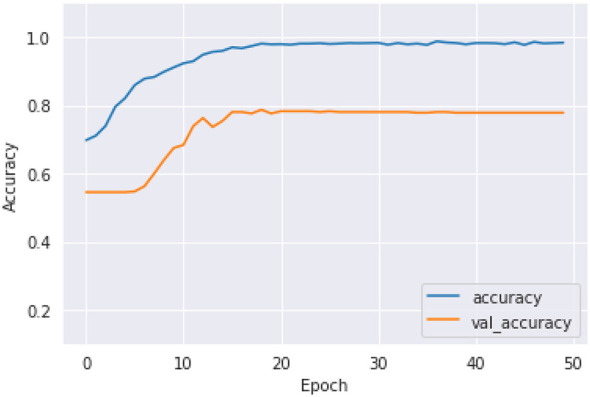
Accuracy of CNN.

The initial stage involves downloading and extracting the data. The photos are then loaded into a TensorFlow dataset. Training utilizes 80% of the data, whereas testing utilizes 20 percent. Consequently, we must establish a “ImageDataGenerator” for the training and testing datasets. The training set generator will also incorporate additional image enhancement techniques, such as zooming and snapshot flipping. Augmentation mitigates the overfitting of networks. The next step is to utilize the generators to load the pictures from the base directory. While loading images, the desired image size is specified. All photos will be scaled to the size chosen. With a batch size of 32, the images are loaded from the directory in batches of 32. We employ pooling with a 2-by-2 filter and a Dropout layer to prevent overfitting in the network. The last layer contains one unit because autistic and non-autistic individuals will be classified. The activation function is Sigmoid since the classification problem involves binary images. Due to the presence of binary classes, the model will be compiled utilizing categorical loss and precision in the future. The performance of ConvNet is depicted in Figures 6, 7. The accuracy of ConvNet is 98.35%, but validation accuracy is not achieved as required, which is 77.80%.

CNN (MHMCNN) is an experimentally devised method. Two distinct feature extraction modules are provided with the facial data set. Image preprocessing operations such as resizing and grayscale conversion are employed prior to feature extraction. After preprocessing, the modified feature descriptor HOG extracts all important and needed picture data. This HOG image descriptor facilitates effective feature extraction. The output feature is subsequently sent to a multichannel CNN. In this case, a 3 × 3 tiny convolution kernel is used for feature extraction in the convolution layer. Small kernel sizes are preferred over fully linked networks in part because they lower computing expenses. The concatenation layer connects three CNNs immediately following their flattening stage in the proposed architecture. In this model, identical input parameters are provided to all three CNN channels, which improves classification performance. Here, three models are coupled simultaneously, and each model independently processes identical input information. The *k*-fold cross-validation approach is performed for the suggested framework to demonstrate its efficacy with a minimal amount of data samples; the value of “*k*” in this instance is 3.

Modified Histogram-based Multichannel CNN (MHMCNN) is an experimentally devised method. Two distinct feature extraction modules are provided with the facial data set. Despite the existence of photographs of varying sizes, resizing and recalling are employed to bring these images to a common size. After preprocessing, the modified feature descriptor HOG extracts all important and needed picture data. This HOG image descriptor facilitates effective feature extraction. The output feature is subsequently sent to a multichannel CNN. In this case, a 3 × 3 tiny convolution kernel is used for feature extraction in the convolution layer. Partially because they reduce computing costs, small kernel sizes are chosen above completely linked networks. The concatenation layer connects three CNNs immediately following their flattening stage in the proposed architecture. In this model, identical input parameters are provided to all three CNN channels, which will increase the detection rate probability. Here, three models are coupled simultaneously, and each model independently processes identical input information. The *k*-fold cross-validation approach is performed for the suggested framework to demonstrate its efficacy with a minimal amount of data samples; the value of “*k*” in this instance is 3. The proposed model is evaluated using *k*-fold cross-validation with *k* = 3. While higher values such as *k* = 5 or *k* = 10 are commonly used for more robust performance estimation, the selection of *k* = 3 in this study is motivated by a trade-off between computational cost and evaluation reliability. The MHMCNN model involves multichannel CNN training, which is computationally intensive, and increasing the number of folds significantly increases training time. Given the moderate dataset size (2,936 images) and balanced class distribution, *k* = 3 ensures sufficient samples in each fold for both training and validation while maintaining stable performance estimates. Experimental observations showed minimal variance across folds, indicating that *k* = 3 provides reliable evaluation in this context.

**Figure 8:**
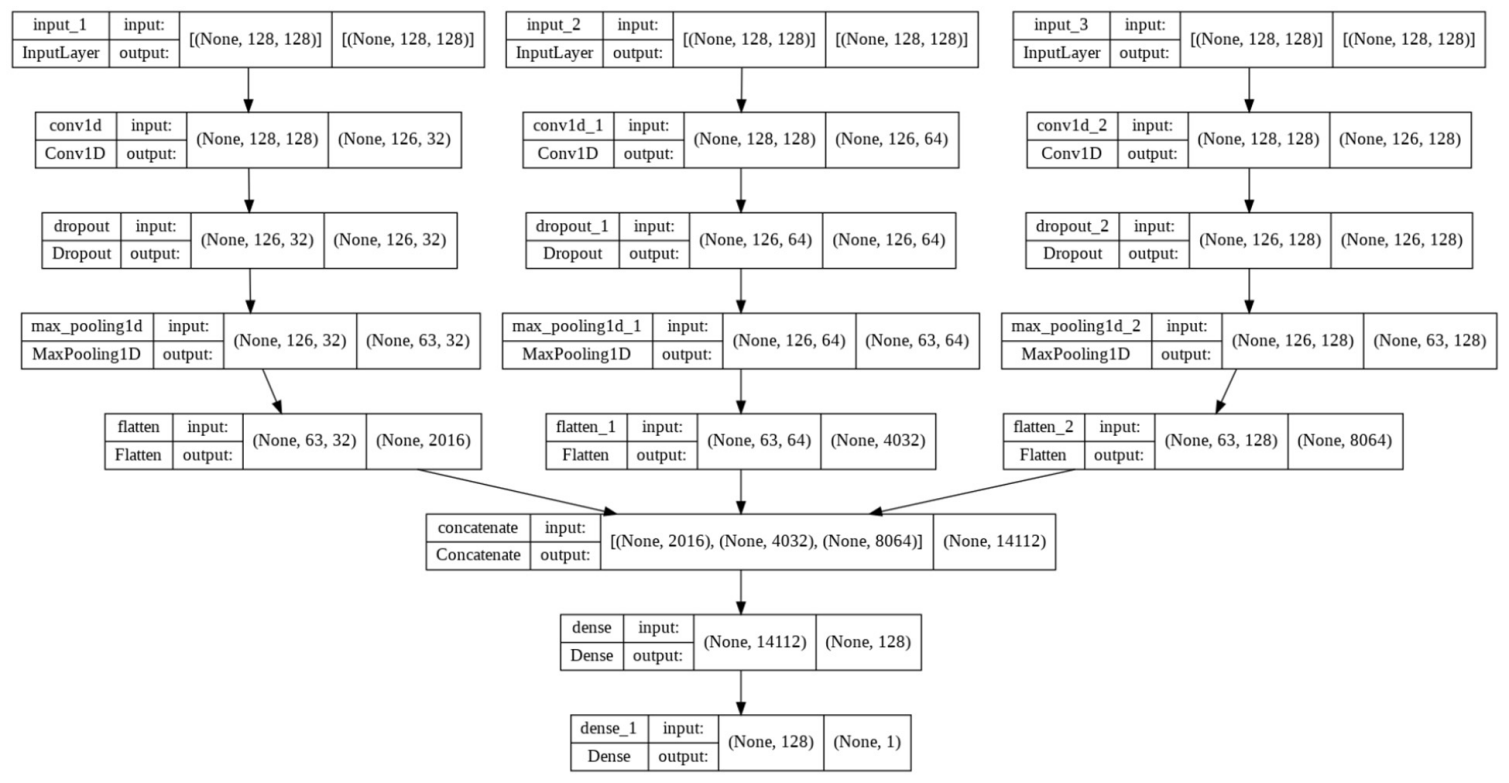
Plot of the multichannel convolution neural network for autism detection using HOG.

**Figure 9:**
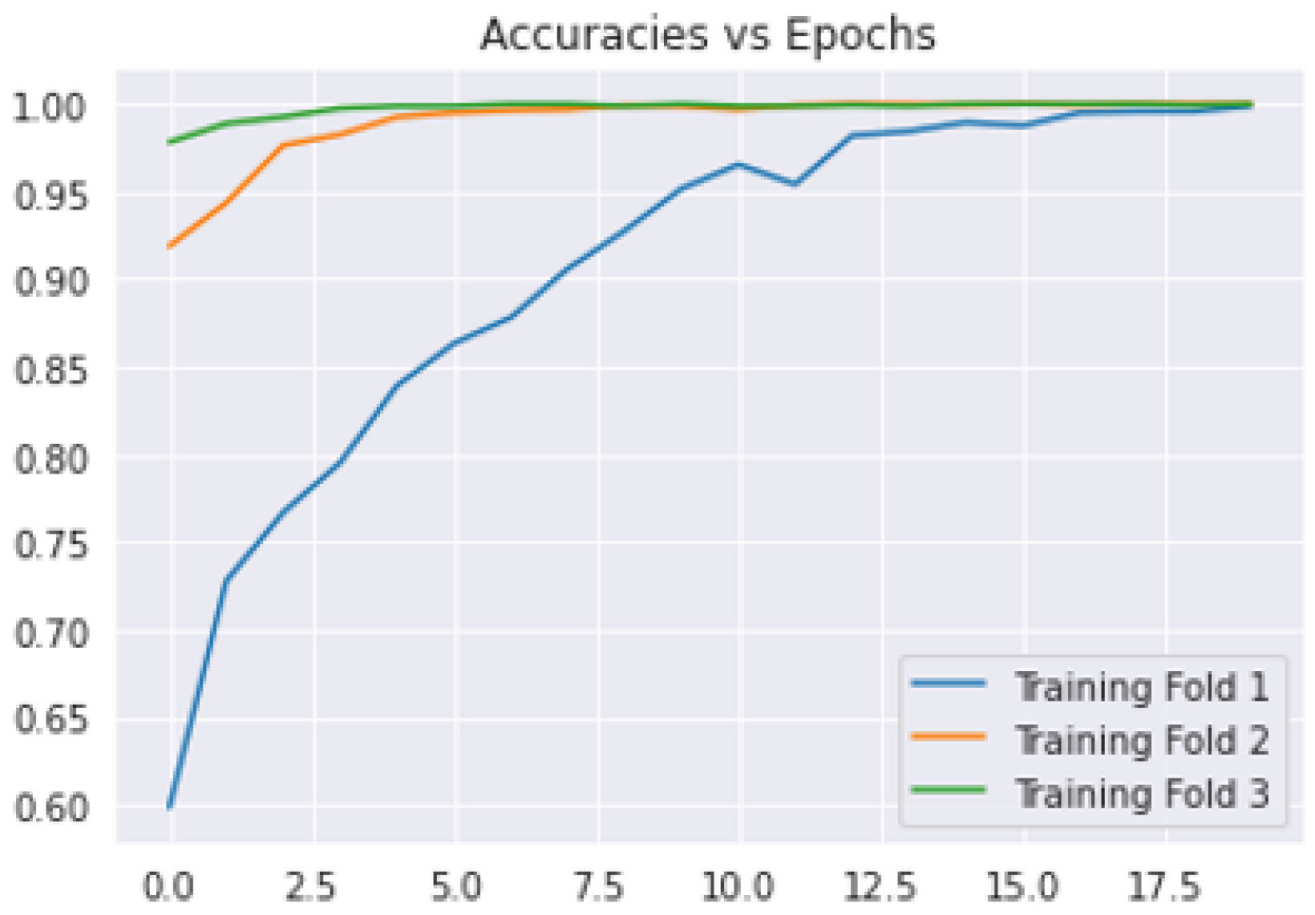
Training accuracy curve of the proposed MHMCNN model over epochs during *k*-fold cross-validation.

**Figure 10:**
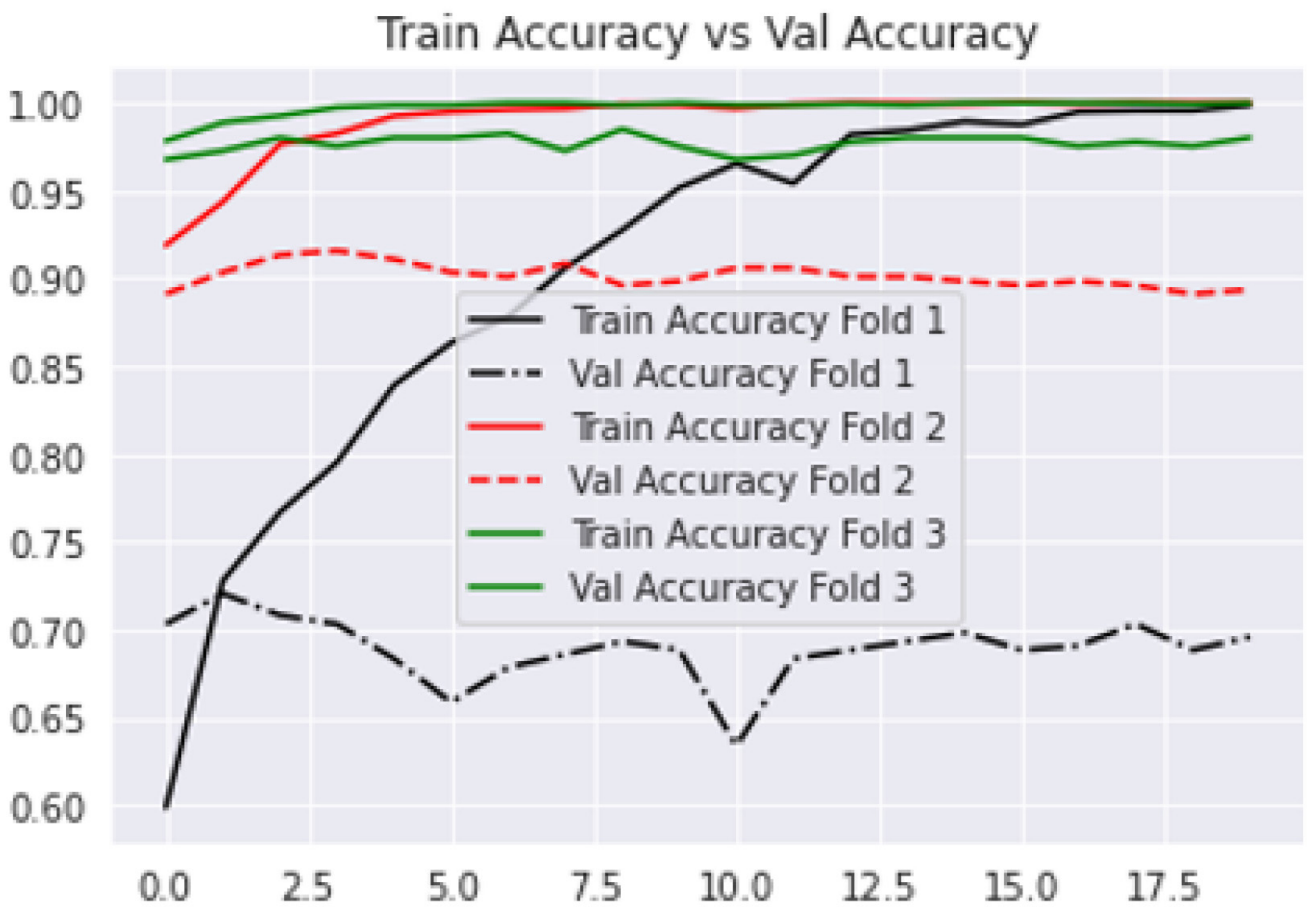
Validation accuracy curve of the proposed MHMCNN model over epochs.

**Figure 11:**
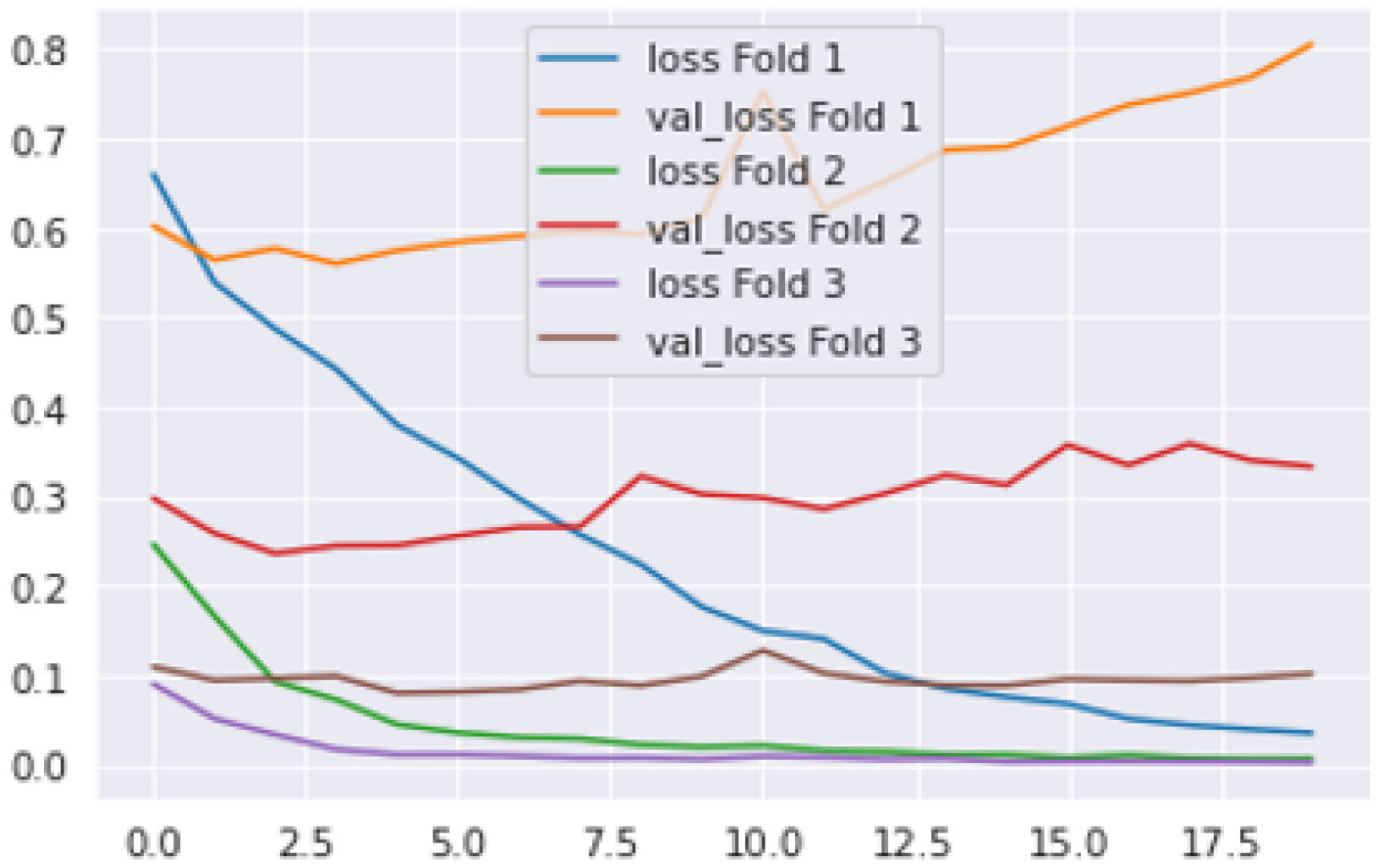
Loss and val loss of proposed model of MHMCNN with *k* = 3.

**Figure 12:**
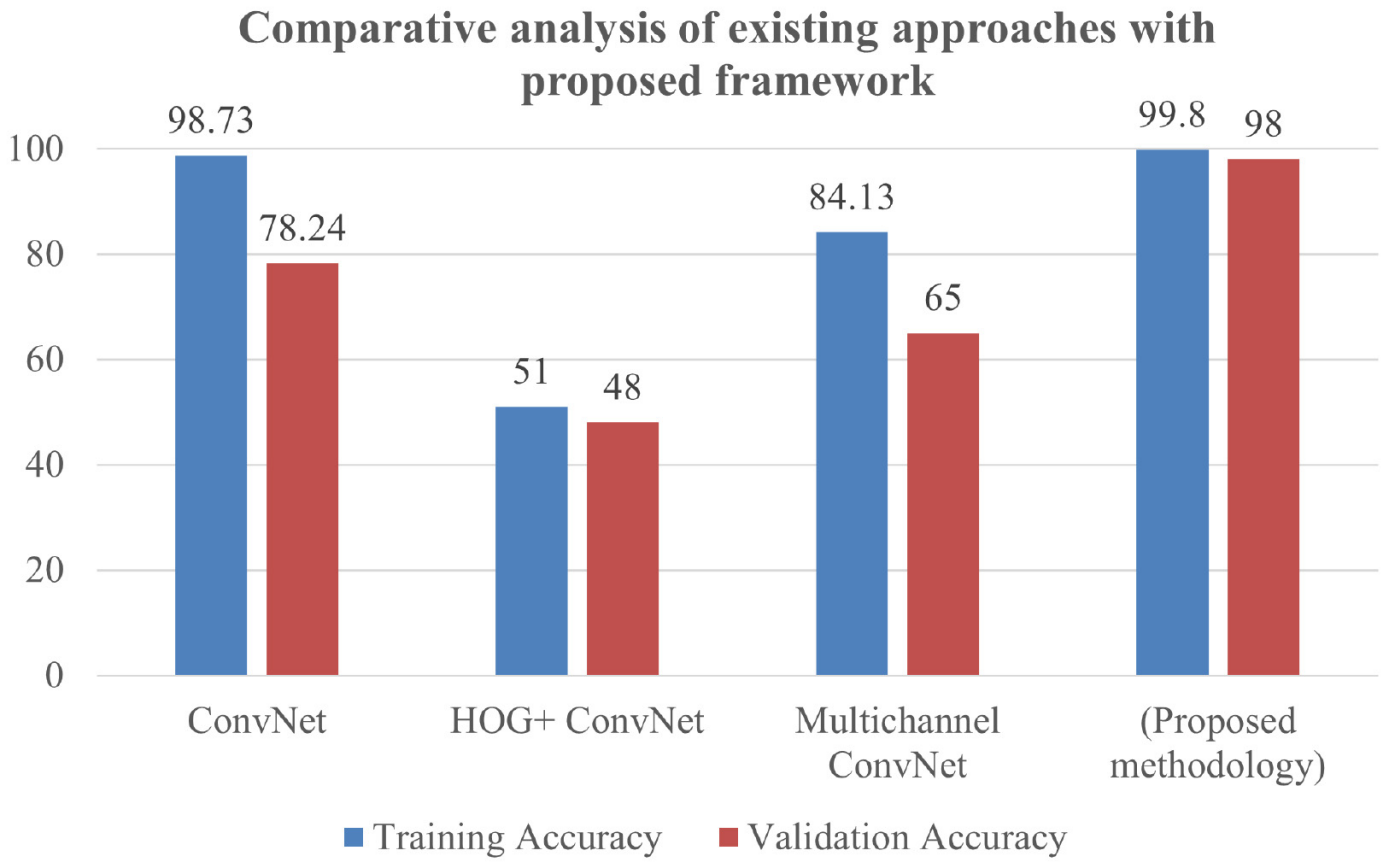
Comparative performance analysis of different models, including ConvNet, HOG+CNN, and the proposed MHMCNN.Comparative performance analysis of ConvNet, HOG+CNN, and the proposed MHMCNN model. The horizontal axis represents the evaluated models, while the vertical axis represents classification accuracy (%).

The detailed performance of the proposed method is well-shown in Figures 8–12.

Figure 9 shows the train accuracy of the proposed model, which shows the proposed MHMCNN model achieved a validation accuracy of 98% and a test accuracy of 96.2%, while reaching a training accuracy of 99.8% during optimization. The figure illustrates a line graph of machine learning model training accuracy with time for three training folds. The *x*-axis shows epochs 0–20 and the *y*-axis shows accuracy levels 0.60–1.0. Three lines show the three training folds. Training Fold 1 is blue, 2 is orange, and 3 is green. The blue line shows accuracy increasing from 0.60 to 0.95 by epoch 17. The orange and green lines start with high accuracy (≥0.90) and stability. The initial epochs bring both lines close to 1.0. The bottom right caption shows the lines by fold.

Wherein Figure 10 shows train as well as validation accuracy after 3 *k*-fold. It is evident the model is much more capable of performing well on validation data with an accuracy of 98%. The image illustrates the trends in the accuracy of a machine learning model over three distinct data folds and 20 epochs. The accuracy levels, which range from 0.6 to 1.0, are represented by the *y*-axis, while the epochs, which range from 0 to 20, are represented by the *x*-axis. Three lines represent the training accuracy for each fold (solid black, red, and green lines), and three lines represent the validation accuracy (dashed lines of the same colors). There are a total of six lines. The validation accuracy lines are more stable, with a delayed increase, while the training accuracy for all folds steadily increases, approaching 1.0 as epochs proceed. An initial decrease in validation accuracy is evident in the initial fold (black lines), which subsequently recovers. The second and third folds (red and green) exhibit cleaner trends and higher validation accuracy, suggesting more consistent model performance. The line patterns are explained by a legend located in the center, which corresponds each hue and style to its corresponding fold and accuracy type.

Figure 11 shows the loss and val loss of the proposed model of MHMCNN with *k* = 3. The image depicts a line graph that illustrates the evolution of the training and validation loss over 20 iterations in three distinct phases of a machine learning model. The *y*-axis displays the loss values, which range from 0 to 0.8. The *x*-axis displays the number of epochs, which ranges from 0 to 20. Six lines comprise the graph. Three solid blue, red, and purple lines indicate the training loss for each crease, while three solid orange, green, and brown lines indicate the validation loss. The loss for Fold 1 (blue), which starts out at a high level and gradually decreases, demonstrates a significant reduction in training loss. Conversely, the validation loss for Fold 1 (orange) exhibits a modest increase in terms of time. A comparable pattern is evident in Fold 2, where the training loss (red) is decreasing and the validation loss (green) is more stable, with a minor rise. Fold 3 demonstrates the lowest initial training loss (purple) and a comparatively consistent validation loss (brown). The color and loss type of each crease are delineated in a legend located at the upper center of the graph. This graph shows a divergence between the training and validation losses in folds 1 and 2, which illustrates the model's loss behavior during training and validation.

**Figure 13:**
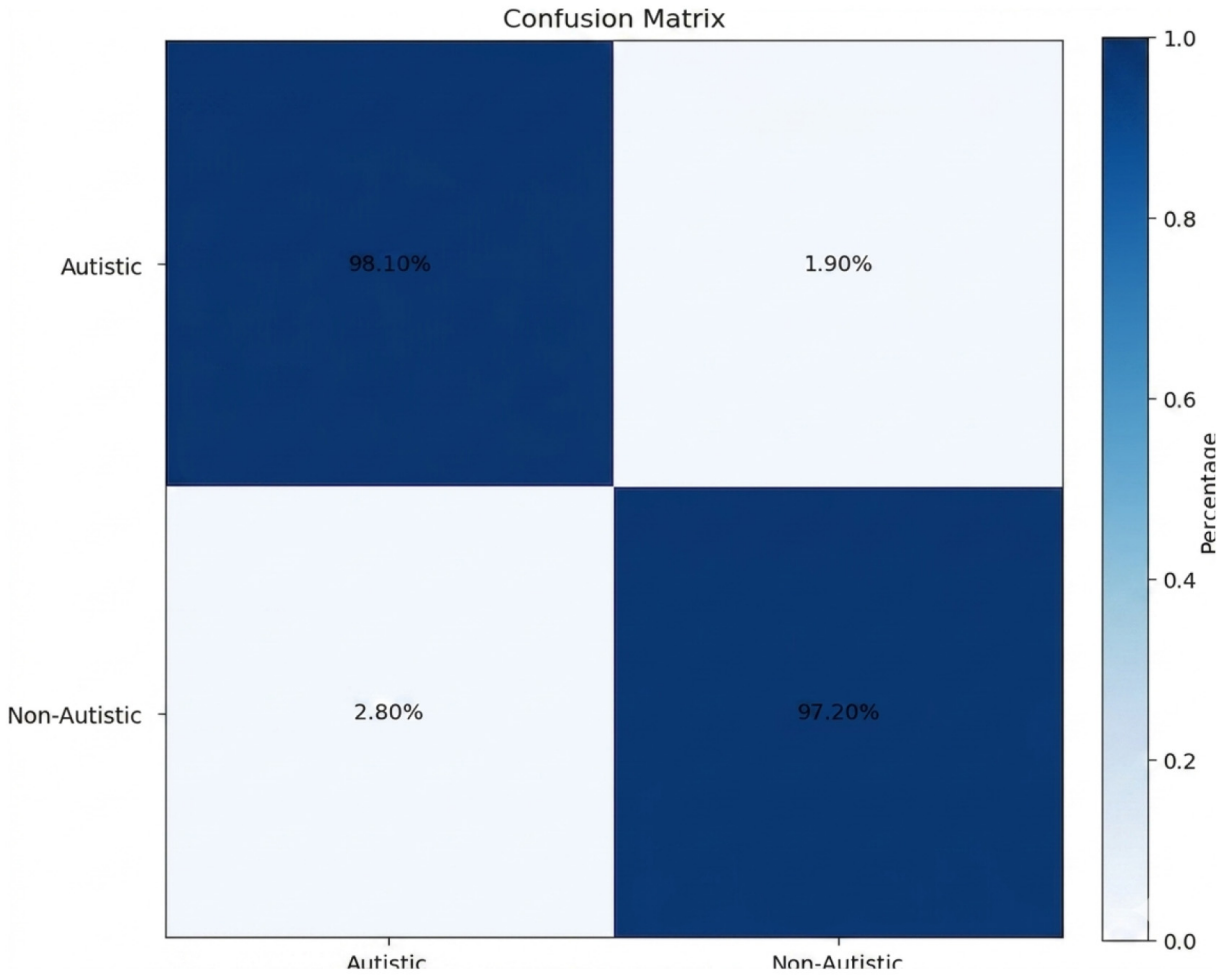
Confusion matrix of the proposed MHMCNN model for ASD classification. The horizontal axis represents predicted class labels, while the vertical axis represents actual class labels.

Figure 13 shows the confusion matrix of the proposed model of autism classification, where in model predicts correct classes with fewer false predictions. The confusion matrix illustrates a model's classification efficacy for “Autistic” and “Non-Autistic.” The matrix has four quadrants. In the upper-left quadrant, 98.10% of the artistic category was accurately categorized. The top-right quadrant accounts for 1.90% of erroneous classifications of non-autistic individuals in the creative domain. Instances in the bottom-left quadrant that were non-autistic were erroneously classified as artistic at a rate of 2.80%, whereas examples in the bottom-right quadrant exhibited an accuracy of 97.20%. The right color bar indicates the percentage scale, including darker blue for elevated values and lighter hues for diminished values. The labels on both axes denote artistic and non-autistic classifications.

The proposed model is designed in collaboration with a modified histogram of gradients with a multichannel CNN model. This section compares the performance of the proposed model with base models. A comparative analysis of base models with the proposed framework is shown in Figure 12. As shown in the figure, the proposed MHMCNN model achieves superior validation and test performance compared with baseline models, indicating improved generalization capability.

#### 5.3.2 Test set evaluation

To assess the generalization capability of the proposed model, a held-out test set comprising 200 images (100 autistic and 100 non-autistic) was used. This dataset was not involved in training or validation phases. On the held-out test set, the proposed MHMCNN model achieved an accuracy of 96.2%, demonstrating its ability to generalize to unseen data. The close alignment between validation and test performance indicates that the model maintains strong generalization with minimal overfitting.

#### 5.3.3 Comparison with state-of-the-art models

Recent advances in facial analysis and medical image classification have widely adopted transfer learning architectures such as VGG16, ResNet50, and EfficientNet, as well as attention-based and transformer-based models. These models benefit from large-scale pretraining and deep feature extraction capabilities. In contrast, the proposed MHMCNN framework is designed as a lightweight hybrid model that integrates handcrafted feature extraction (HOG) with multichannel CNN learning (Chen et al., 2024; Bahathiq et al., 2022). Unlike transfer learning models, which rely on pretrained weights from large datasets such as ImageNet, the proposed model is trained from scratch on the target dataset, making it computationally efficient and adaptable to domain-specific data. While direct experimental comparison with these architectures is beyond the scope of the current study, existing literature suggests that transfer learning models achieve strong performance in facial classification tasks. However, they often require higher computational resources and longer training times. The proposed MHMCNN offers a trade-off between computational efficiency and classification performance, achieving competitive accuracy (98% test accuracy) with reduced complexity. Future work will include benchmarking the proposed model against state-of-the-art architectures, including attention-based CNNs and transformer models, to further validate its effectiveness.

## 6 Conclusion and future research

This study presents a machine learning-based framework for ASD detection. Children typically exhibit this condition between the ages of three and five. The current diagnostic and classification models for autism spectrum disorders necessitate a significant amount of information and time. In comparison to the standard single-channel models, the suggested multi-channel CNN demonstrated superior diagnostic performance. This study begins by presenting the issue at hand, which is identifying high-functioning autism through traditional diagnostic approaches and machine learning models. The next step presents a comprehensive literature review. Conventional autism detection studies are scarce, and the number considered for this purpose is even smaller than the other methods. The proposed MHMCNN framework achieved a validation accuracy of 98% and a test accuracy of 96.2%, demonstrating its effectiveness for ASD classification using facial image analysis. We can enhance the effectiveness of the proposed model by incorporating additional techniques. The suggested model could enhance autism recognition by incorporating facial expression and audio data. Future research should explore this further. The proposed study introduces a new framework for using machine learning to identify autism spectrum disorder in children with a high assessment accuracy of 98%. Still, several limitations and considerations exist. First, the use of only one dataset from Kaggle raises concerns about its size, diversity, and representations. To ensure the framework's performance in different populations, studies with large and diverse datasets on different populations should evaluate its effectiveness. Furthermore, the model's ability to generalize beyond its training dataset remains uncertain. To prove the model's robustness, evaluations with independent datasets from different sources are needed. Moreover, ethical considerations about dataset bias and model interpretation should be mentioned. Deep learning models such as multichannel CNNs can be very complex and thus hard to interpret. Therefore, we need tools to explain model decisions. Moreover, adding a complementary data modality beyond facial expression, that is, speech patterns as well as behavioral evaluations, may further support the accuracy, versatility, and functionality of the framework. Finally, the key steps are the real-world deployment and clinical authentication of forensic studies. University and clinical integration may precisely assess the long-term ramifications of early ASD detection on intervention as well as the specific quality of life. By taking into account these shortcomings, we can enhance the credibility of this work and establish effective frameworks for early ASD examination.

## Data Availability

The original contributions presented in the study are included in the article/supplementary material, further inquiries can be directed to the corresponding authors.
